# Ultra-rapid, sensitive and specific digital diagnosis of HIV with a dual-channel SAW biosensor in a pilot clinical study

**DOI:** 10.1038/s41746-018-0041-5

**Published:** 2018-08-17

**Authors:** Eleanor R. Gray, Valérian Turbé, Victoria E. Lawson, Robin H. Page, Zara C. Cook, R. Bridget Ferns, Eleni Nastouli, Deenan Pillay, Hiromi Yatsuda, Dale Athey, Rachel A. McKendry

**Affiliations:** 1 London Centre for Nanotechnology, University College London, 17–19 Gordon Street, London, WC1H 0AH UK; 2 Division of Medicine, University College London, Gower Street, London, WC1E 6BT UK; 3 OJ-Bio Ltd, Biomedicine West Wing, International Centre for Life, Times Square, Newcastle-upon-Tyne, NE1 4EP UK; 4 Division of Infection and Immunity, University College London, London, WC1E 6BT UK; 5 Clinical Microbiology and Virology, University College London NHS Foundation Trust, London, W1T 4EU UK; 6 Africa Health Research Institute, KwaZulu Natal, South Africa; 7 Japan Radio Co. Ltd, Saitama, 356-8510 Japan

**Keywords:** HIV infections, Biosensors, Infectious-disease diagnostics

## Abstract

Despite widened access to HIV testing, around half of those infected worldwide are unaware of their HIV-positive status and linkage to care remains a major challenge. Current rapid HIV tests are typically analogue risking incorrect interpretation, no facile electronic data capture, poor linkage to care and data loss for public health. Smartphone-connected diagnostic devices have potential to dramatically improve access to testing and patient retention with electronic data capture and wireless connectivity. We report a pilot clinical study of surface acoustic wave biosensors based on low-cost components found in smartphones to diagnose HIV in 133 patient samples. We engineered a small, portable, laboratory prototype and dual-channel biochips, with in-situ reference control coating and miniaturised configuration, requiring only 6 µL plasma. The dual-channel biochips were functionalized by ink-jet printing with capture coatings to detect either anti-p24 or anti-gp41 antibodies, and a reference control. Biochips were tested with 31 plasma samples from patients with HIV, and 102 healthy volunteers. SH-SAW biosensors showed excellent sensitivity, specificity, low sample volumes and rapid time to result, and were benchmarked to commercial rapid HIV tests. Testing for individual biomarkers found sensitivities of 100% (anti-gp41) and 64.5% (anti-p24) (combined sensitivity of 100%) and 100% specificity, within 5 min. All positive results were recorded within 60 s of sample addition with an electronic readout. Next steps will focus on a smartphone-connected device prototype and user-friendly app interface for larger scale evaluation and field studies, towards our ultimate goal of a new generation of affordable, connected point-of-care HIV tests.

## Introduction

The United Nations Sustainable Development Goals herald a major commitment by the world to halt the spread of HIV/AIDS by 2030.^1^ Yet despite huge advances in testing and antiretroviral therapy, around half of the estimated 37 million people living with HIV in the world do not know their HIV-positive status,^2,3^ leading to late presentation complicated by significant immunosuppression, opportunistic infections and related mortality,^4^ and contributing to the spread of HIV in the community.^5,6^ Pilot studies have shown that widening access to testing, through placing testing sites in locations such as community centres and money transfer shops, is key to reaching sections of the population who would not normally encounter HIV testing through traditional services.^7^ Moreover, self-testing is now acceptable, feasible, and has been made legal in some countries with internet and smartphone-based apps under development to facilitate testing and linkage to care.^8,9^ Patients can only engage with care if they are aware of their HIV status. Diagnosis early in infection carries significant benefits of increased life-expectancy by 10 years and facilitates access to treatment, for example, reducing the risk of mother to child transmission to under 1% whereas late diagnosis carries an increased risk of onwards transmission and death. Therefore widening access to diagnosis is a priority, alongside new strategies to link patients to care.^5,10–12^

Worldwide, the most common rapid HIV tests are based on lateral flow immunochromatography, similar to pregnancy tests. They are low cost but suffer from a number of key limitations. Firstly, although they are rapid compared to gold-standard laboratory-based tests, most require longer than 10 min to perform and cannot be run within a typical appointment with a general practitioner in the UK.^13^ Interpretation is not trivial, as even healthcare workers who daily administer rapid tests can exhibit repeated operator errors that lower sensitivity and specificity, such as reading tests before the stated time period necessary for accurate interpretation has passed, and mis-reading of faint bands.^14,15^ Most rapid tests in current use are entirely analogue by design, without automated electronic data capture, risking data loss and errors in manual entry of data. Only a few are compatible with an automated reader, which removes the need for user interpretation but doesn’t reduce the time to result or automate long-term recording of results into electronic medical records.^16,17^ There is no automated follow-up of patients, leading to potential for loss through the care cascade.^10,18^ Moreover, the advent of self-testing leads to an increase in first-time users without extensive training. A study showed that only 26% of naïve, first-time self-testers correctly interpret weakly reactive tests.^15^

Smartphones have come to the fore in recent years as tools to facilitate access to and widen capabilities of rapid diagnostic devices, using the inbuilt sensors and computational power of the device, or their ability to wirelessly link results to public health systems.^19^ The smartphone camera can be used in conjunction with algorithms to analyse results of lateral flow test strips, and the battery of phones has been used to power a microfluidic dongle attachment for dual HIV-syphilis tests.^20–23^ Key research challenges include compliance with target product profile guidelines for suitable characteristics of an HIV rapid test set out by the Programme for Appropriate Technology in Health (PATH) such as a closed system, and fewer than three steps.

Here we report on the development of a digital diagnostic device based on shear horizontal surface acoustic wave (SH-SAW) biosensors and a clinical pilot study of this biosensor to detect HIV with 133 patient samples. The principle of SH-SAW dates back to Lord Rayleigh in 1885, with the first sensing application reported in 1979,^24^ and the first application as a biosensor in 1983.^25^ For decades, a key challenge facing the field for biosensing applications was the compatibility of liquid samples with SH-SAW electrical components.^26^ A breakthrough came through the engineering of microfabricated SH-SAW chips with a glass-polymeric capping layer to protect interdigitated electrodes, making them compatible with complex biological samples, without the need to use complex microfluidics which can clog.^27^ The SH-SAW biosensors described herein utilise microelectronic components to detect multiple patient HIV antibodies without additional complexity of washing or component labelling—technology which has the potential to advance to the next generation of ASSURED biosensors for disease diagnostics.^28^ SH-SAW sensors are suitable for economic mass manufacture, costing *ca*. $1.50 for each disposable biochip and can quantitatively detect the binding of proteins to their surface.^29^ They have been used with a wide range of sample types in a variety of media to detect, for example, bacteria in micro-volumes,^30^ cellular interactions,^31^ DNA^32^ and have also been used as biosensors for the detection of antibodies to viral pathogens in blood.^33^ In addition, unlike lateral flow tests, the readout provided by SH-SAW biosensors is entirely electronic, thereby avoiding the risk of incorrect interpretation of the test result. The absence of need for interpretation of the test result, simplicity of use, low cost and user-friendly presentation of the test result are key points that form part of the target product profile for HIV self-tests set out by PATH.^34^ The electronic readout can be carried out by a smartphone app (prototype in development), which could help to widen access to testing given the ubiquity of smartphones, particularly amongst hard to reach groups such as young people.^35^ The app could also be used to initiate follow up appointments, closing a gap in the care cascade.^9^

Herein we report on a number of significant technical developments, which builds on our previous proof of concept work using purified antibodies to HIV in buffer samples^36^; (i) A new dual-channel biochip with in-situ reference to improve specificity. Our previous study utilised two manually functionalized independent biochips; a detection biochip and reference biochip.^36^ Although this study highlighted the potential of the technology, the use of separate biochips posed challenges as each biochip was constructed on a separate piezoelectric crystal and functionalized manually. This could lead to inter-chip variation, and failure to accurately control for non-specific effects when samples or volumes differ across two biochips. Here, the biochips have been significantly improved with multiple channels on a single biochip, with an in-situ reference channel to account for non-specific signals, for example, from sample viscosity; (ii) Miniaturized device configuration requiring smaller sample volumes. The SH-SAW biosensors used in this study require just 6 µl sample in comparison to the previous device which required 20 µl, bringing the volume within the ‘optimistic’ target range specified by PATH (10 µl).^34^ A smaller sample makes the test less invasive for those who find it difficult to produce large or multiple blood drops (such as neonates or the elderly), and a miniaturized device uses less raw material, lower costs, and less waste; (iii) Biochip functionalization via inkjet printing amenable to mass manufacture. Automation of biochip preparation removes variation in chip preparation steps, increases homogeneity of chips, and therefore consistency of results in comparison to manually prepared biochips; (iv) Detection of multiple HIV biomarkers to increase specificity and sensitivity. Our previous study focused on detection of anti-p24 antibodies alone. Here we also detect anti-gp41, which is the first antibody biomarker of HIV infection, opening up the possibility of earlier diagnosis of HIV. We investigated the sensitivity and specificity of both biomarkers, and combined sensitivity and specificity when both biomarkers are used, since case studies have reported the absence of anti-gp41 antibodies among a small number of patients^37,38^; (v) Pilot clinical study with 133 patient samples. Our previous study largely focused on recombinant proteins in buffer solution, and testing of one HIV positive patient sample and one healthy volunteer, insufficient to determine the clinical sensitivity and specificity. By contrast, here we test 133 samples, 31 from patients with HIV, and 102 healthy volunteers; (vi) Analysis of biochip variation. We present head to head results from two biochips for each HIV-positive sample; (vii) Benchmarking to leading commercial rapid tests. We compared the performance of SH-SAWs to leading rapid index tests in terms of sensitivity, specificity, sample volume, time to result and electronic data capture.

## Results

### Dual-channel biochips with an in-situ reference channel to increase specificity

**Fig. 1:**
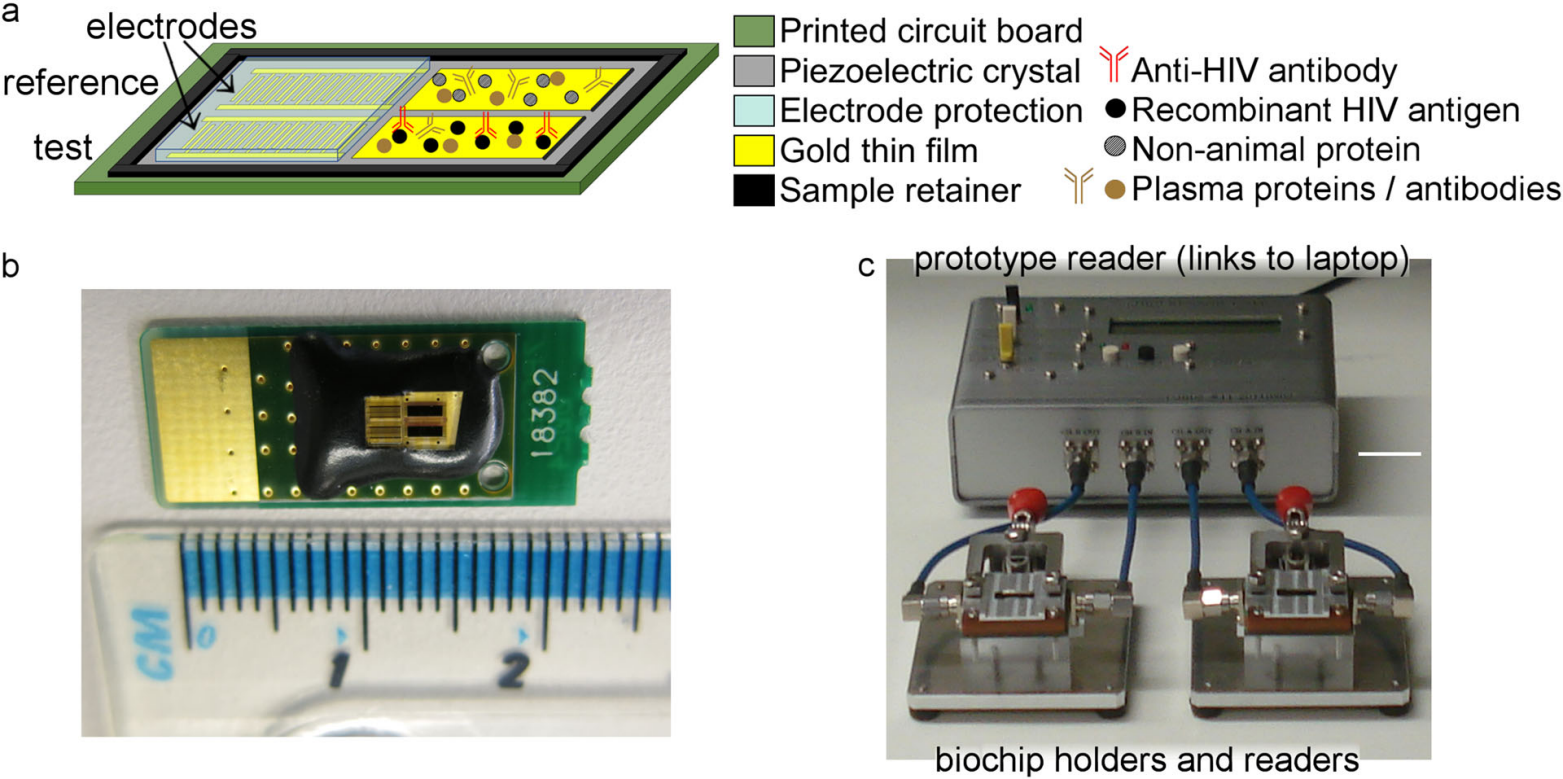
SH-SAW biosensor and biochips. **a** Key characteristics of dual-channel biochips. **b** Dimensions of disposable laboratory-use biochips. Channels are surrounded by black resin, with electrodes under glass protection. **c** Laboratory-based biochip holders and prototype reader (biochips in **b** are beneath metal covers). This was the reader used in this study. White scale bar shows 2 cm

The underlying principles of SH-SAW biosensors and their application to the development of rapid HIV tests to detect analytes in biological samples was described in the refs.^39^^36^ In brief, SH-SAW biochips comprise a sensing area with waterproof electrodes at one end. This sensing area is sensitive to biological binding events. Figure 1a–c shows the key components used in this study. A schematic of the SH-SAW biochips is shown in Fig. 1a and Supplementary Figure 1, with the two channels and different coatings of each channel represented by symbols. The actual biochips used are shown in Fig. 1b, where the channels and electrodes are surrounded by a black resin to contain liquid samples. Electrodes are protected by a glass lid. An image of a prototype reader used for the experiments described in this paper is shown in Fig. 1c, which was connected to a laptop.

The biochip reference channel was coated with an artificial synthetic protein that is designed to be completely unlike any human or animal protein (non-animal protein, NAP), and only non-specific binding and a signal change due to the viscosity of the sample placed on the chip will be recorded on this channel. The signal from this reference channel can be used to compensate for non-specific effects in the ‘antibody-detection’ channel by calculation (i.e., ‘antibody-detection’ channel minus reference channel). This permits SH-SAW biochips to be used with a wide range of sample types with varied viscosities such as blood or serum.

Two antibody detection channel coatings were used to capture either anti-gp41 or anti-p24. Anti-gp41 is usually the first antibody to appear after HIV infection and therefore a key immune marker of HIV infection (Supplementary Figure 2 and ref. ^40^). Inkjet printing automates biochip channel preparation, marking an important step towards an efficient and cost effective manufacturing, and improving on the previous generation of biochips which were manually functionalized.^36^

**Fig. 2:**
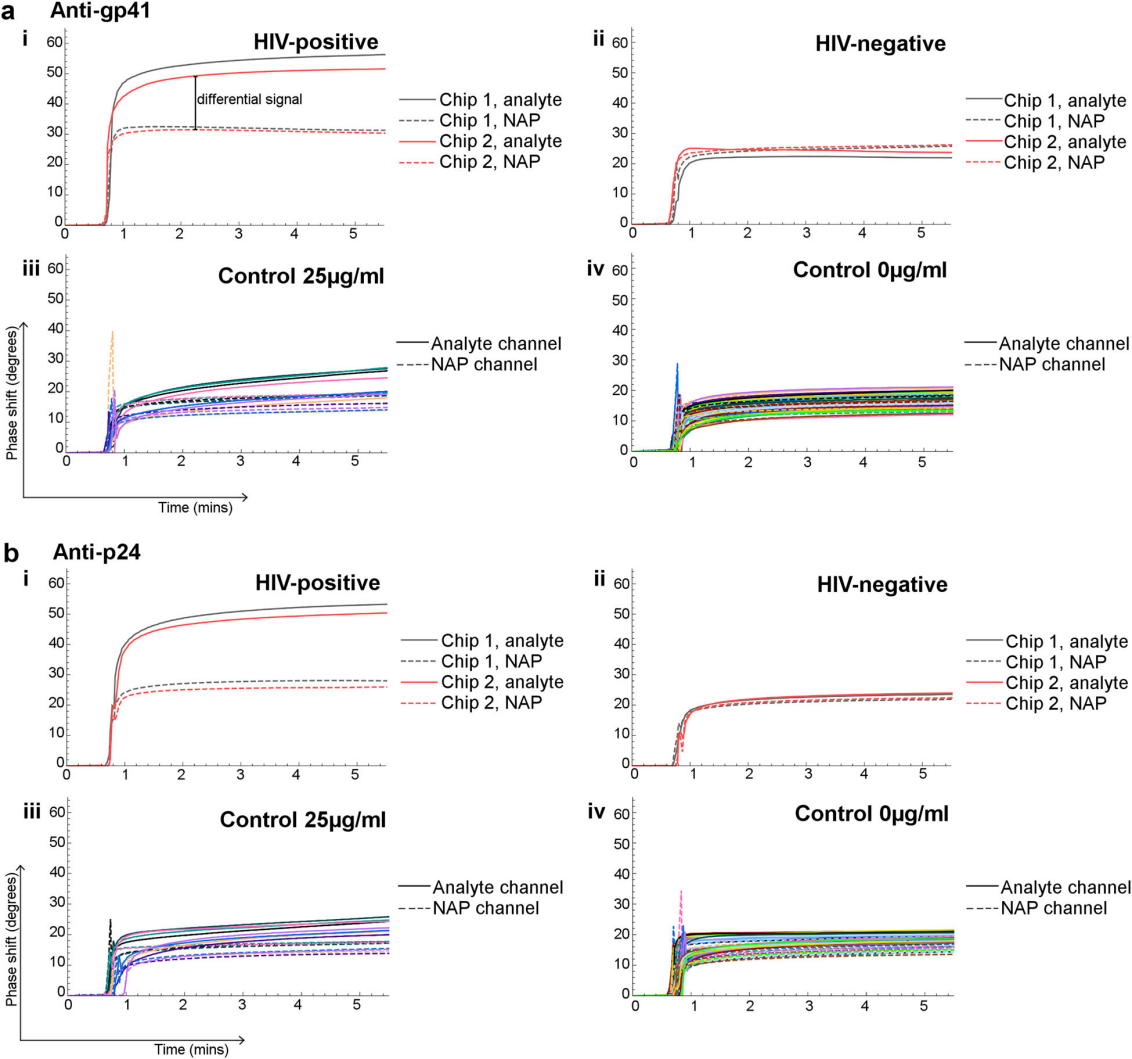
Raw signal readout from anti-gp41 and anti-p24 biochip individual runs. Shown are reference (dashed lines) and analyte (continuous) channels for **a** anti-gp41 and **b** anti-p24 biochips; (i) a single HIV-positive sample, two biochips; (ii) a single healthy donor sample on two biochips; for calibration samples at (iii) 25 μg/ml; and (iv) 0 μg/ml. The differential signal is indicated in a (i) (analyte minus reference channels)

The SH-SAW chips were initially stored unfunctionalized for several months at room temperature. After biochips were prepared by functionalization, they were stored at 4 °C (with desiccant) for one to two weeks until analysis. During a sample run, biochips were equilibrated in 6 μl Tris-buffered saline with 0.5% Tween-20 for 30 s to ensure that they were responding normally after placement in the biochip holders. After 30 s, the buffer was removed and 6 μl sample added. An example of a raw data readout from a single HIV-positive and a single HIV-negative sample run on two separate biochips each detecting anti-gp41 is shown in Fig. 2a, i, ii respectively, and for anti-p24 in Fig. 2b, i, ii respectively. The differential signal generated by subtracting the reference channel from the antibody detection channel gives the assay output, reported as differential SH-SAW reading (Fig. 2a, i). Pooled confirmed negative donor samples were spiked with purified human anti-HIV antibody at concentrations ranging between 0 and 100 μg/ml to validate biochip preparation and assay viability. Raw data readouts from runs with 25 and 0 µg/ml antibody are shown for anti-gp41 in 2a, iii and 2a, iv respectively, and for anti-p24 in 2b, iii and 2b, iv respectively. All calibration data are shown in Supplementary Figure 3. From these spiked calibration samples, preliminary limits of detection can be calculated for anti-p24 of 22.2 µg/ml, and anti-gp41 of 25.5 µg/ml.

### Pilot study of clinical sensitivity and specificity

**Fig. 3:**
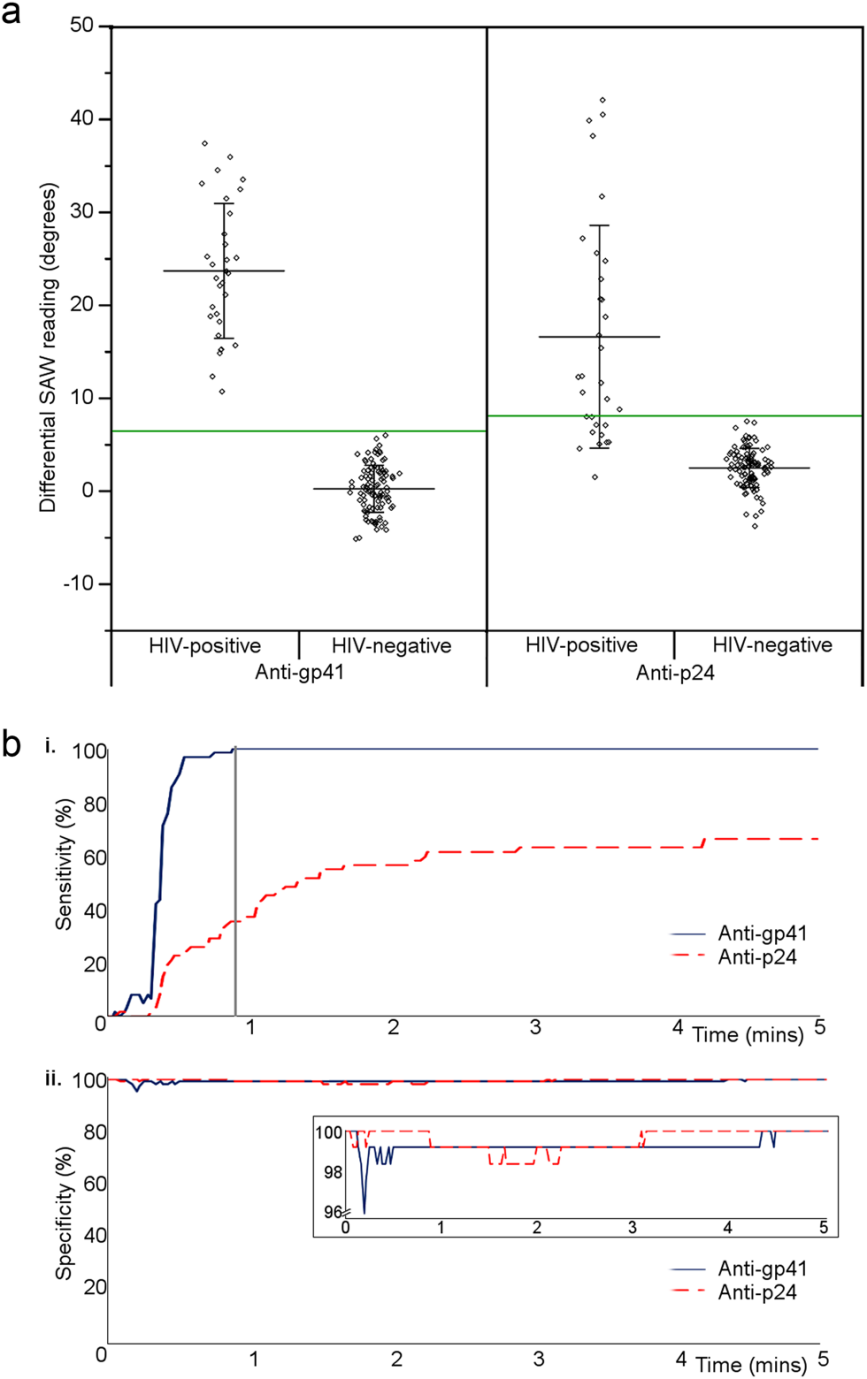
Combined biochip data. **a** Differential signals for 31 HIV-positive (duplicate averages) and 102 healthy volunteer samples on anti-gp41 and anti-p24 biochips. Means are shown with standard deviations for each group. Green lines, positive threshold cut-offs. **b** (i) Time-resolved sensitivity for HIV-positive samples. By 53 s (grey marker) all positives gave a reactive result. (ii) Time-resolved specificity data. After 5 min, all negative samples gave negative results. Inset: Enlarged *Y*-axis view

Assays were run for 31 patient samples in duplicate, 98 healthy donors once, and four healthy donors twice, all for anti-gp41 and anti-p24 biochips. The differential signal for each run is plotted in Fig. 3a separately for anti-gp41 and anti-p24 readings. A sample was considered positive if the differential reading values for either or both of the anti-gp41 and anti-p24 biochips was above the threshold, and negative only if both readings were below the thresholds (Fig. 3a, green lines, and Supplementary Table 1). For anti-gp41, the threshold was defined as the mean plus four times the standard deviation of the mean of the differential signal from repeated pooled negative samples, and for anti-p24, plus five times this value; these values were chosen to position all negative results below the cut-off. Average HIV-positive test results above this threshold were considered positive, and under this threshold, negative. All of the samples from patients with HIV gave a true positive average reading for anti-gp41 detection, giving a sensitivity of 100% for this part of the test alone (Fig. 3a). For anti-p24 detection, 20 samples gave average true positive readings, and 11 gave an average false negative reading, giving a sensitivity of 66.1%. Overall, therefore, the combined sensitivity was 100% as defined by a positive reading for one or both anti-gp41 and anti-p24 biomarkers (Table 1 and Supplementary Table 1). Samples were run in duplicate to assess biochip variation (Supplementary Figure 4); results from the two runs were not significantly different (*p* = 0.99, *t*-test).

**Table 1:** Collated results for the SH-SAW biochips on confirmed HIV-positive and HIV-negative samples

	HIV positive samples			HIV negative samples			Sensitivity (%)	Specificity (%)
	Total	TP	FN	Total	TP	FN		
Anti-gp41	31	31	0	102	102	0	100	100
Anti-p24	31	20	11	102	102	0	64.5	100
Combined	31	31	0	102	102	0	100	100

Sensitivity and specificity were calculated for the average results for either anti-gp41 or anti-p24 individually and in combination. If a sample gave a positive result for either analyte, it was deemed positive, but must be negative for both analytes to be considered negative

*TP* true positive, *FN* false negative, *FP* false positive, *TN* true negative

Neither anti-p24 nor anti-gp41 markers were detected in any of the healthy donor samples, giving a specificity of 100% of detection for both antibodies (Fig. 3a).

### Time to result

In order to assess the speed of the assay, the time for the differential signal to cross the cut-off threshold was plotted for anti-gp41 or anti-p24 (Fig. 3b). All of the positive samples had given a true-positive readout for anti-gp41 within 60 s (Fig. 3b, i, grey line). After five minutes, all the negative donor samples had remained negative (Fig. 3b, ii, shown in duplicate inset on an enhanced scale).

### Comparison to commercial rapid HIV tests

The samples from the patients with HIV were from patients with viral loads of at least 5,000 copies/mL, obtained at the stage of discard by the laboratory, and had been kept at 4 °C for three weeks. It is unclear what effect this might have on the samples although degradation, rather than the opposite, is probable. In order to validate the results obtained from the antibody portion of the test, the samples were therefore also run on two commercially available point-of-care (POC) index tests; the Alere HIV-1/2 Ag/Ab Combo, and the OraQuick Advance. These two were in use in the UK at the time of our study, and the OraQuick Advance is also sold as an HIV self-test in the US, though other, faster rapid tests are now available (for example, the Pasante Insti). The results of these rapid tests with the samples used in this study are shown in Table 2. All HIV enzyme immunoassay-positive samples gave clear positive antibody readings on both the Alere HIV-1/2 Ag/Ab Combo and the OraQuick Advance tests. None of the HIV-positive samples gave a positive p24 antigen reading using the Alere HIV-1/2 Ag/Ab Combo test, but a positive antigen result was not expected given that the samples are from patients who have probably seroconverted. The two commercial rapid tests both detect anti-gp41, which was also positive in all samples by the SH-SAW assay. These results verify the validity of the results for anti-gp41 obtained using the SH-SAW biochips.

**Table 2:** A comparison of test characteristics and results for the OJ-Bio SH-SAW test and two commercial POC HIV-1 index tests

**Sample**	**OJ-Bio SH-SAW biosensor**	**OraQuick Advance HIV-1/2**	**Alere determine HIV-1/2 Ab/Ag Combo**
Antibody/Antigen (Ab/Ag)	Ab	Ab	Ab
Biomarker targets	Anti-gp41 Anti-p24	Anti-gp41	Anti-gp41
All HIV-positive samples	+	+	+
All healthy donor samples	–	–	–
Pooled donor plasma	–	–	–
Volume of sample required (μl)	6	2.5	50
Time to result (min)	5	20	20
Electronic data capture and connectivity	Inbuilt	None	None

Shown is a summary of results from the pilot clinical study in comparison to results obtained from two currently available POC tests in the UK for samples used in this study. Ab, antibody; Ag, antigen. See Table 1 and Supplementary Table 1 for numbers and results of samples tested on the SH-SAW biosensors

## Discussion

Our findings highlight the potential of SH-SAW biosensors with dual-channel biochips to detect the presence of anti-HIV antibodies at clinically-relevant levels, with 100% sensitivity and 100% specificity, within very short (60 s) timescales at low (6 μl) sample volumes. With further development into a fully user-friendly, sample-preparation free smartphone-connected format, SH-SAW biosensors have potential as a foundation for the next generation of low cost (*ca*. $1.50 for disposable components), rapid, POC screening tests. These performance characteristics align to the World Health Organisation’s target product profile for upcoming HIV rapid tests that have the potential to facilitate the transformation of testing, enabling it to stand apart from traditional healthcare settings and trained workers, and empowering patients themselves in non-traditional settings (Supplementary Table 2 and http://www.idc-dx.org/resources/target-product-profile-hiv-self-test). An optimal POC test requires no sample preparation steps, minimal input and interpretation required from the user, would link the patient directly into healthcare services with wireless connectivity and yet would operate with the same sensitivity and specificity as those currently available for professional healthcare workers.

The 100% sensitivity for anti-gp41 and 100% specificity achieved in this small pilot study is promising. Next steps will focus on testing whole blood with a larger number of patient samples, the development of a final cartridge format and smartphone connected prototype, amenable to field usage, and longevity studies of biochip shelf-life at a variety of different temperatures and time-scales. A final cartridge format will be required to omit the buffer stabilisation steps used herein, and the encasement of the SH-SAW biochip entirely in plastic to ensure that the chip is not flooded by an average drop of sample (20–50 µl). The detection of two antibodies either on a single test channel or separately on two ensures that sensitivity will remain high in sporadic cases of patients with low reactivity to gp41 antigen.^37,38^ During the course of this project multi-channel biochips have been designed which means that the two tests described here could be combined on one biochip, and eventually multiplexed with additional channels. Ideally, a channel would be dedicated to a target of acute HIV infection, such as p24 antigen or RNA, to permit diagnosis of those at an early stage after transmission. Alternatively, tests for other blood-borne diseases could be incorporated to enable a general first-line screening tool, as a multiplexed blood-borne panel (see, for example ref. ^23^).

This small study used samples that were at the stage of discard from the diagnostic laboratory and had been stored for three weeks between receipt and discard. It was not possible to know at which stage of infection the patients were at the time of sampling, though as they had viral loads of at least 5000 copies/mL it is not probable that they were taking effective antiretroviral therapy. It is possible that they were recently diagnosed, however, all had seroconverted and were reactive by two third generation rapid antibody tests, making this scenario unlikely. The enzyme immunoassay used at UCLH to initially determine the status of the samples does not discriminate between antigen-positive and antibody-positive, and the two rapid tests used in this study detect anti-gp41 and not anti-p24. There is therefore no guarantee that these samples were assuredly anti-p24 positive. Usage of many more negative samples with an appropriate sample size calculation would also permit refinement of the cut-off value, as well as a more accurate characterisation of the specificity of the SH-SAW biosensor. Nevertheless, the levels of sensitivity achieved remain promising. For the purposes of this initial pilot study with patient samples, we note that we have used only plasma samples taken from routine submissions for viral load testing at University College London Hospital (UCLH) at the point of discard, when they had been already separated into plasma and cellular matter for storage. Future studies will be performed in a larger trial using freshly drawn whole blood and potentially other sample types such as saliva.

During this study we used a laboratory prototype SH-SAW biochip reader connected to a laptop (Fig. 1c). A prototype consumer unit (handheld reader controlled by a smartphone app and disposable cartridge, Supplementary Figure 5) has been made, which contains the same SH-SAW biochip components as the laboratory prototype, and the ability to transfer test results via Bluetooth to the smartphone. The SH-SAW biochip in the disposable cartridge is hidden under the plastic coating, is designed for use with whole blood without sample preparation, and is self-contained and entirely disposable which limits the potential for contamination. Challenges of moving to the next stage of development will include: (i) Moving the test outside the laboratory environment, where temperature, humidity, and dust levels will be variable; (ii) Working directly with freshly drawn whole blood, with samples of variable haematocrit; (iii) Testing of SAW biochips with low levels of haemolysis, which can occur subsequent to finger prick blood draw; (iv) Addition of samples without pipettes directly from patients, with non-quantified and differing volumes of blood; (v) Training requirements for usage of the test by non-laboratory staff. Prototype consumer units in development utilise a variety of approaches to handle whole blood samples. One approach that has been investigated is the use of a membrane to retain red blood cells, while allowing the liquid fraction of plasma to migrate through to the SAW biochip. This takes place inside the test cartridge, with limited opportunity for interference or complexity for the user. We aim to use feedback from preliminary trials with inexperienced users to refine instructions, training and the user interface. Parallel to these will be the general challenges of implementation in settings where patients come for POC HIV testing, and integration of the technology into existing clinical pathways. Close partnerships between the product development and manufacturing teams, as well as clinical collaborators and end-users during development will ensure that the technology developed is suited to the specific needs of end-users. Future work will focus on testing the performance of this prototype in the following areas that will be key for clinical adoption: evaluate our prototype using performance panels representative of the existing diversity of HIV subtypes worldwide; evaluate performance with different types of specimens that will aid use in the field (i.e., finger prick whole blood and saliva); test an extended number of patients prospectively in a field study by healthcare workers and also potentially self-testing. Data transfer through an electronic programme such as an app permits future connectivity of results to public health data systems

To close, our findings lay the foundation for a new paradigm of digital diagnostic technology that combines all of the benefits of current rapid HIV tests based on lateral flow (cost, time to result, simplicity of use) with the additional benefits of electronic data capture, no user-interpretation, geo-located connectivity, and the ability to link the patient into care, supporting faster access to treatment and counselling for those who test both positive and negative.

## Methods

### Recombinant antigen and purified antibody

Recombinant p24 and gp41 were obtained from Capricorn Bioproducts (USA) and Fitzgerald Industries (USA) respectively. Purified human anti-HIV-1 antibody was obtained from ImmunoDX (USA). All reagents were stored and processed according to the manufacturer’s recommendations.

### SH-SAW Biochip preparation

SH-SAW biochips are described in the ref. ^39,41^ and biochip manufacture was performed essentially as described in the ref. ^36^ Briefly, biochips were based on a quartz crystal (36°Y-cut 90°X-propagation) operating at a frequency of 251.5 MHz. Gold interdigitated transducers were evaporated onto the crystal, consisting of 80 finger pairs with an aperture of 1 mm. Two nanometre thin film titanium, then 90 nm thin film gold was evaporated in between the interdigitated transducers, which were protected from liquid by a glass lid an epoxy walls, as described in the ref. ^27^ Biochips used here were a later version with dual channels. Biochip preparation was performed essentially as described in the ref. ^36^ Briefly, biochips were cleaned with 2% Hellmanex™, functionalized using 4 mg/ml dithiobis(succinimidyl propionate) and channel-specific capture protein using an automated Musashi Jet Spotter platform. For this study, dual channel biochips were prepared with non-animal protein (NAP, G-Biosciences, USA) on the reference channel and recombinant gp41 or p24 antigen on the detection channel. Biochips were dried under a stream of N_2_, and stored at 4 °C with desiccant until use.

### SH-SAW assays

SH-SAW chips were made in batches of 200–400 chips; quality control criteria required an insertion loss in air of between 21 and 22 dB. SH-SAW biochips with a dual channel (reference and test channel) were used with 6 µl sample for each biochip run, which is the maximum load of the biochips. The biochip surface is covered with approximately 4 µl, after which the measurement is insensitive to changes in volume. Phase shift, which indicates levels of bound biological matter, was recorded every second for 30 s after buffer addition, and then for up to 5 min after buffer removal and sample addition. The change in phase shift attributed to sample antibody binding was calculated from the sample (detection) channel after subtracting the signal from the reference NAP channel. A quality control calibration curve was run prior to sample testing to ensure functional biochip preparation for the batch using 0, 10, 25 and 100 μg/ml purified human anti-gp41 or anti-p24 antibody (diluted in negative pooled plasma).

### Point-of-care (POC) tests

All tests were run as per the manufacturers’ instructions for stored plasma samples, and readout results were combined from two independent operators.

### Plasma samples

Pooled HIV-negative plasma samples, and individual negative samples from screened commercial donors were obtained from Lee Biosolutions (USA) in January 2016. These samples were screened for HIV by FDA-approved methods. HIV-negative samples were also taken after informed consent was obtained from healthy volunteers of UCL staff and students between November to December 2015. HIV-positive samples were collected from consecutive patients attending UCLH HIV services over a period of a month within September to October 2014 who had a viral load of over 5000c/mL (using an in-house assay), and were anonymized and retained after clearance for discard from UCLH diagnostic laboratory. Aside from the viral load, no further eligibility criteria were imposed. All samples were aliquotted upon receipt and stored at −80 °C until use (up to 18 months).

### Cut-off calculation

Repeat negative samples (pooled negative donor) were run to calculate an upper readout value under which 99.99%, or over 99.99%, of the negative samples would be contained (the mean plus four times and five times the standard deviation of the results from pooled negative samples for anti-gp41 and anti-p24 respectively). This was chosen as a cut-off threshold. Average HIV-positive test values above this limit were considered true positives, and values below this, false negatives.

### Ethics statement

The HIV enzyme immunoassay-positive samples were anonymized and had clearance for discard from the UCLH diagnostic laboratory. They were collected as part of the ICONIC project, approved by the Ethical Committee NRES Committee London - Surrey Borders HRA, Research Ethics Committee (REC) London Centre Study title: InfeCtion respONse through vIrus genomiCs (ICONIC) REC reference:13/LO/1303. HIV viral load was above 5000 c/mL. Samples obtained from UCL staff and students were taken after full informed consent was given. The study was reviewed by UCL Ethics Board and given study number 6109/001.

### Data availability

The datasets generated and analysed during the current study analysis is available from the corresponding author on reasonable request.

### Code availability

Custom Mathematical code used for analysis is available from the corresponding author on request.

## Electronic supplementary material

Supplementary Material

## Electronic supplementary material

**Supplementary information** accompanies the paper on the *npj Digital Medicine* website (10.1038/s41746-018-0041-5).

## References

[1] United Nations Development Programme. Sustainable Development Goals: Goal 3: Good Health and Well-Being. (2016). Available at: http://www.undp.org/content/undp/en/home/sustainable-development-goals/goal-3-good-health-and-well-being/targets/. (Accessed: 22nd September 2017)

[2] UNAIDS. Global AIDS update 2016. 1–16 (2016).

[3] Global Statistics. (2017). Available at: https://www.hiv.gov/hiv-basics/overview/data-and-trends/global-statistics. (Accessed: 26 January 2018).

[4] Croxford S (2017). Mortality and causes of death in people diagnosed with HIV in the era of highly active antiretroviral therapy compared with the general population: an analysis of a national observational cohort. Lancet Public Health.

[5] Cohen MS (2011). Prevention of HIV-1 infection with early antiretroviral therapy. N. Engl. J. Med.

[6] Marks G, Crepaz N, Janssen RS (2006). Estimating sexual transmission of HIV from persons aware and unaware that they are infected with the virus in the USA. AIDS.

[7] Yin, Z. et al. HIV in the United Kingdom: 2014 Report. 1–51 (2015).

[8] Pant Pai, N. et al. Supervised and unsupervised self-testing for HIV in high- and low-risk populations: a systematic review. *PLoS Med*. **10**, e1001414–14 (2013). 10.1371/journal.pmed.1001414 PMC3614510 23565066

[9] Pant Pai, N. et al. Will an unsupervised self-testing strategy for HIV work in health care workers of South Africa? A cross sectional pilot feasibility study. *PloS one* **8**, e79772–9 (2013). 10.1371/journal.pone.0079772 PMC3842310 24312185

[10] Rice B (2014). Trends in HIV diagnoses, HIV care, and uptake of antiretroviral therapy among heterosexual adults in England, Wales, and Northern Ireland. Sex Transm. Dis..

[11] Gardner EM, McLees MP, Steiner JF, del Rio C, Burman WJ (2011). The spectrum of engagement in HIV care and its relevance to test-and-treat strategies for prevention of HIV infection. Clin. Infect. Dis..

[12] Bor J, Herbst AJ, Newell ML, Bärnighausen T (2013). Increases in adult life expectancy in rural South Africa: valuing the scale-up of HIV treatment. Science.

[13] NHS Choices. GP appointments—The NHS in England—NHS Choices. http://www.nhs.uk/NHSEngland/AboutNHSservices/doctors/Pages/gp-appointments.aspx. Accessed 22nd September 2017.

[14] Wolpaw BJ (2010). The failure of routine rapid HIV testing: a case study of improving low sensitivity in the field. BMC Health Serv. Res..

[15] Peck RB (2014). What should the ideal HIV self-test look like? A usability study of test prototypes in unsupervised HIV self-testing in Kenya, Malawi, and South Africa. AIDS Behav..

[16] Johnson S, Cushion M, Bond S, Godbert S, Pike J (2015). Comparison of analytical sensitivity and women’s interpretation of home pregnancy tests. Clin. Chem. Lab. Med..

[17] Tomlinson C, Marshall J, Ellis JE (2008). Comparison of accuracy and certainty of results of six home pregnancy tests available over-the-counter. Curr. Med. Res. Opin..

[18] Galvan FH, Brooks RA, Leibowitz AA (2004). Rapid HIV testing: issues in implementation. AIDS Patient Care STDS.

[19] Xu X (2015). Advances in smartphone-based point-of-care diagnostics. Proc. IEEE.

[20] Laksanasopin T (2015). A smartphone dongle for diagnosis of infectious diseases at the point of care. Sci. Transl. Med..

[21] Mudanyali O (2012). Integrated rapid-diagnostic-test reader platform on a cellphone. Lab Chip.

[22] Kim D (2018). Enzyme-free nucleic acid amplification assay using a cellphone-based well plate fluorescence reader. Anal. Chem..

[23] Joh DY (2017). Inkjet-printed point-of-care immunoassay on a nanoscale polymer brush enables subpicomolar detection of analytes in blood. Proc. Natl Acad. Sci. USA.

[24] Wohltjen H, Dessy R (1979). Surface acoustic wave probes for chemical analysis. II. Gas chromatography detector. Anal. Chem..

[25] Roederer JE, Bastiaans GJ (1983). Microgravimetric immunoassay with piezoelectric crystals. Anal. Chem..

[26] Länge K, Rapp BE, Rapp M (2008). Surface acoustic wave biosensors: a review. Anal. Bioanal. Chem..

[27] Kogai T, Yoshimura N, Mori T, Yatsuda H (2010). Liquid-phase shear horizontal surface acoustic wave immunosensor. Jpn J. Appl. Phys..

[28] Mabey D, Peeling RW, Ustianowski A, Perkins MD (2004). Diagnostics for the developing world. Nat. Rev. Micro.

[29] Berkenpas E, Bitla S, Millard P, da Cunha MP (2004). Pure shear horizontal SAW biosensor on langasite. IEEE Trans. Ultrason Ferroelectr. Freq. Control.

[30] Anisimkin VI (2015). Plate acoustic wave sensor for detection of small amounts of bacterial cells in micro-litre liquid samples. Ultrasonics.

[31] Saitakis M, Gizeli E (2011). Acoustic sensors as a biophysical tool for probing cell attachment and cell/surface interactions. Cell Mol. Life Sci..

[32] Tsortos A, Grammoustianou A, Lymbouridou R, Papadakis G, Gizeli E (2015). The detection of multiple DNA targets with a single probe using a conformation-sensitive acoustic sensor. Chem. Commun..

[33] Lee HJ (2009). Surface acoustic wave immunosensor for real-time detection of hepatitis B surface antibodies in whole blood samples. Biosens. Bioelectron..

[34] PATH. *Target Product Profile: HIV Self-Test Version 4.1: A White Paper on the Evaluation of Current HIV Rapid Tests and Development of Core Specifications for Next-Generation HIV Tests*. 1–60 (2014).

[35] Estcourt CS (2017). The eSexual Health Clinic system for management, prevention, and control of sexually transmitted infections: exploratory studies in people testing for Chlamydia trachomatis. Lancet Public Health.

[36] Turbé V (2017). Towards an ultra-rapid smartphone- connected test for infectious diseases. Sci. Rep..

[37] Brown P, Merline JR, Levine D, Minces LR (2008). Repeatedly false-negative rapid HIV test results in a patient with undiagnosed advanced AIDS. Ann. Intern. Med..

[38] Stekler JD (2009). HIV testing in a high‐incidence population: is antibody testing alone good enough?. Clin. Infect. Dis..

[39] Brookes J, Bufacchi R, Kondoh J, Duffy DM, McKendry RA (2016). Determining biosensing modes in SH-SAW device using 3D finite element analysis. Sens. Actuators B.

[40] Tomaras GD (2008). Initial B-cell responses to transmitted human immunodeficiency virus type 1: virion-binding immunoglobulin M (IgM) and IgG antibodies followed by plasma anti-gp41 antibodies with ineffective control of initial viremia. J. Virol..

[41] Kogai T, Yatsuda H (2014). Liquid-phase membrane-type shear horizontal surface acoustic wave devices. Sens. Mater..

