# Supplementary material for: Ultra-rapid, sensitive and specific digital diagnosis of HIV with a dual-channel SAW biosensor in a pilot clinical study
Source: NPJ Digit Med. 2018 Aug 17;1:35. doi: 10.1038/s41746-018-0041-5 (PMC6550230; doi:10.1038/s41746-018-0041-5)
Supplement: Supplementary file 1 — Supplementary Material [file 41746_2018_41_MOESM1_ESM.pdf]

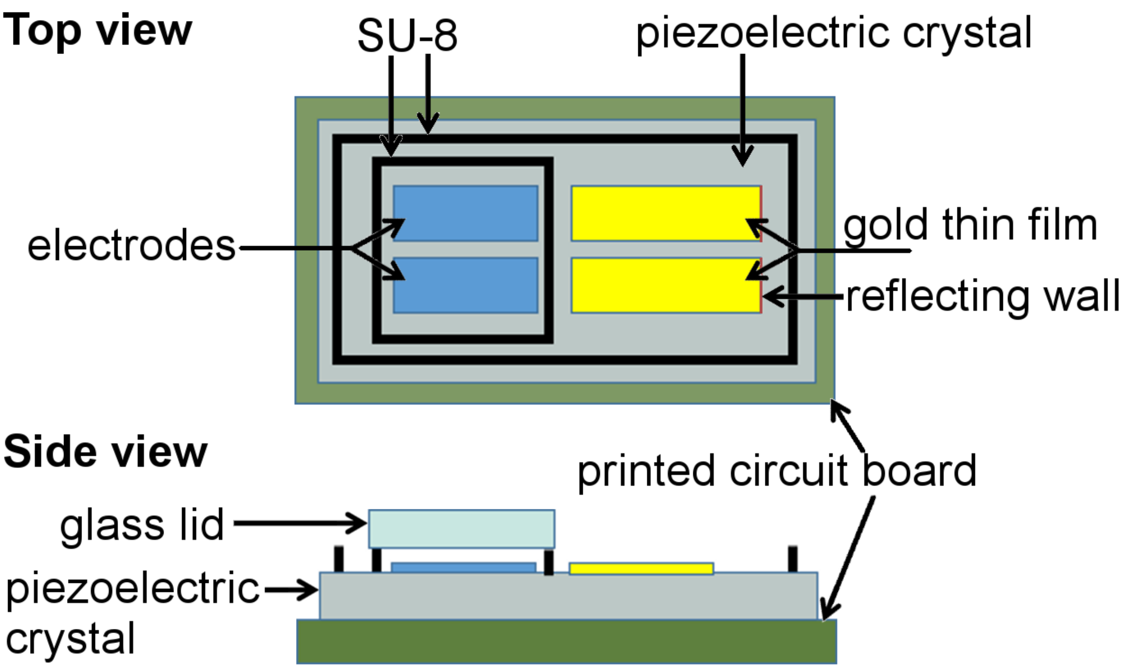

Supplementary Figure 1. Structural characteristics of the SAW biochips, shown in cartoon format.

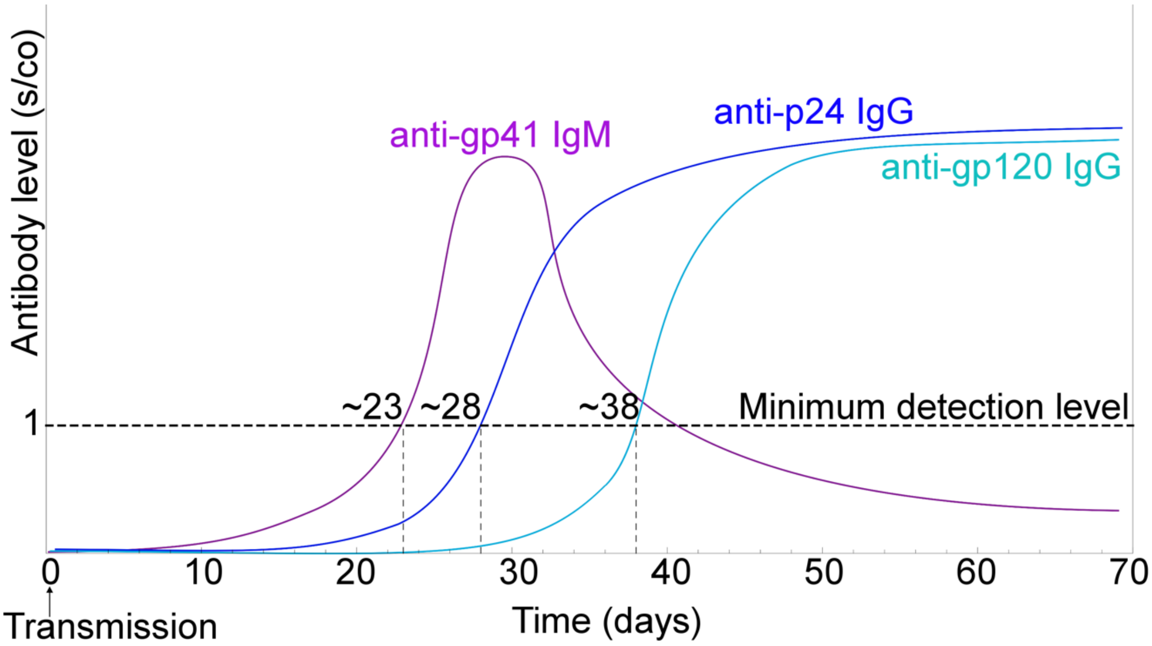

Supplementary Figure 2. Schematic of the appearance of antibodies to different proteins of HIV, spanning from transmission to seroconversion, which is generally within 2-3 months of transmission. Adapted from [1] and references therein.

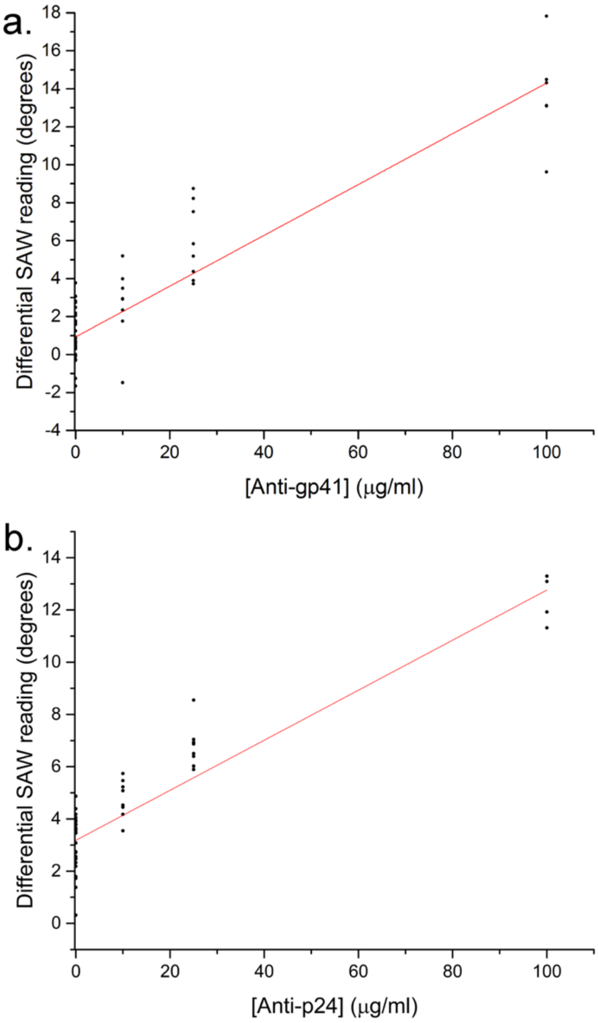

Supplementary Figure 3. Calibration samples comprising pooled negative donor samples spiked with purified human (a) anti-gp41 or (b) anti-p24 antibody at 0, 10, 25 and 100µg/ml. Data shown were gathered across three independent experiments, with at least four data points per concentration per experiment. From these experiments, a calculation can be made for a preliminary value for the limit of blank (LoB) and limit of detection (LoD). $LoB = mean_{blank} + 1.645*SD_{blank}$, and $LoD = LoB + 1.645*SD_{low\ concentration\ sample}$ [2]. LoD anti-p24: 22.2ug/ml; LoD anti-gp41: 25.5ug/ml. As a quantitative output from the assay presented in this study is not required as the qualitative presence of HIV antibodies at any level is indicative of infection, the limit of quantification can be considered to be the same as the LoD.

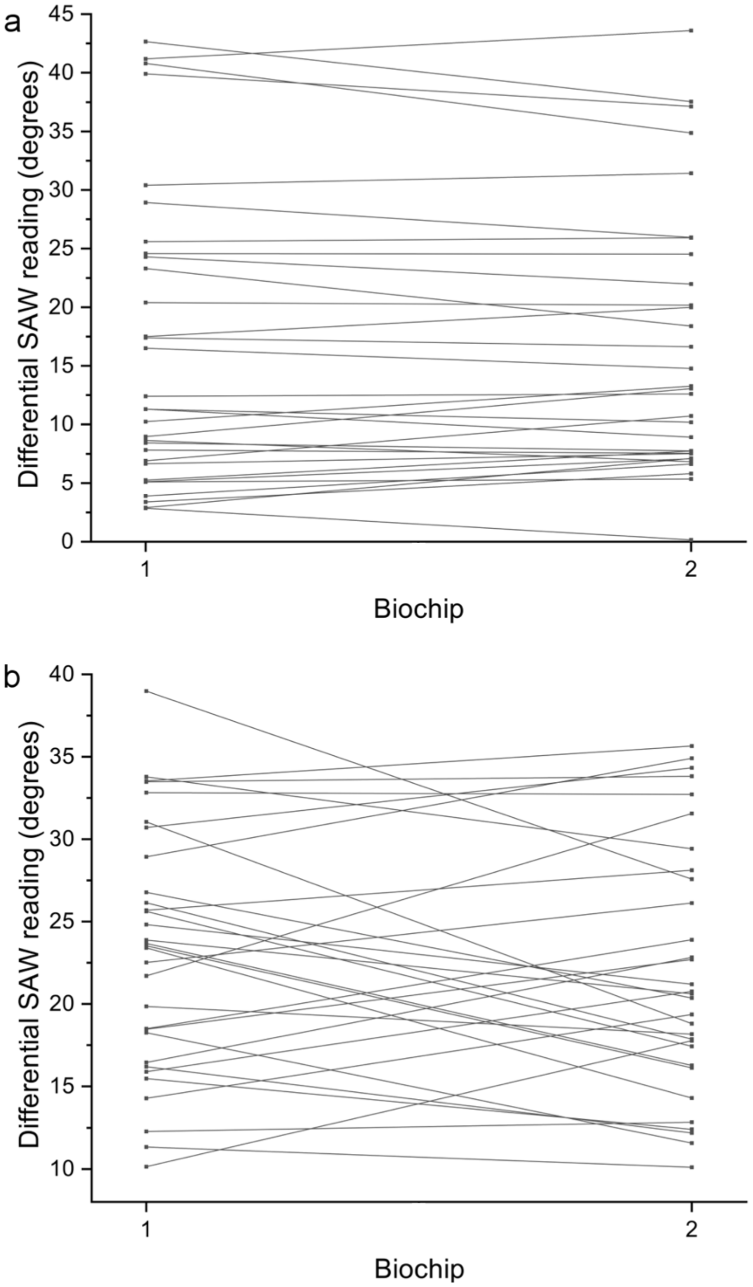

Supplementary Figure 4. Results from duplicate testing of 31 HIV-positive samples on (a) anti-gp41 and (b) anti-p24 detection biochips. Samples were added to the two biochips at the same time in the same rig. See also Supplementary Figure 3 for data from calibration samples.

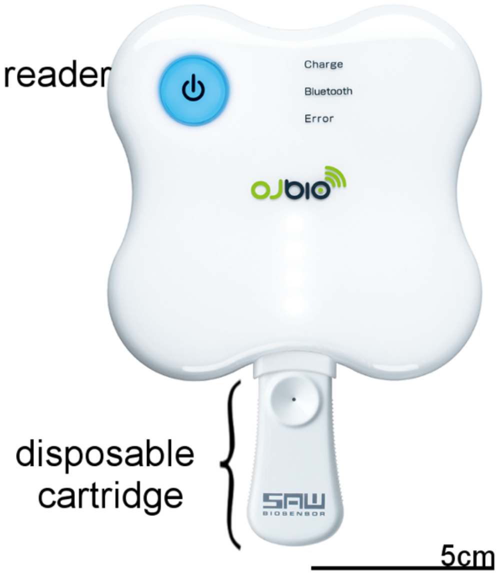

Supplementary Figure 5. Prototype SH-SAW biosensor comprising a reader with inserted disposable cartridge (including biochip), controlled by a smartphone app. The disposable cartridge-enclosed biochip limits contaminants.

	HIV positive samples		HIV negative samples	
	SAW biosensor reading		SAW biosensor reading	
	Average	CI (95%)	Average	CI (95%)
**Anti-gp41**	23.7	21.1 to 26.3	0.194	-0.296 to 0.683
**Anti-p24**	16.6	12.4 to 20.8	2.53	2.13 to 2.93

Supplementary Table 1. Average endpoint readings derived from the SAW biosensors from confirmed HIV-positive and HIV-negative samples for anti-gp41 and anti-p24 individually. CI, confidence interval. All values given to 3 significant figures.

Qualification	Minimal	Optimal	OJ-Bio SAW Biosensor
Setting	Non-healthcare facility or home-testing		Decentralised settings and home testing feasible
Results	Clearly interpretable as reactive, non-reactive or invalid with minimal instructions	Clearly interpretable as reactive, non-reactive or invalid with no instructions	Clear electronic read-out of reactive, non-reactive or invalid with no instructions or user interpretation
Clinical sensitivity	>99%	>99%	Preliminary results: 100%
Clinical specificity	>98%	>99%	Preliminary results: 100%
Field sensitivity	>70%	>90%	Not yet tested
Field specificity	>90%	>98%	Not yet tested
Training	No training required. User able to conduct test correctly after brief review of instructions. Support available in case of questions		Not yet tested but simple one step reaction with no complex sample preparation or wash step
Time to result	< 20 minutes	< 5 minutes	< 5 minutes
Stability of result	≥ 40 minutes	≥ 24 hrs	Recorded electronically
Safety precautions	Closed, self-contained system; unprocessed sample transfer only; no open handling of bio-hazardous material; if lancet required for specimen collection, retractable lancet. Biohazard information should be clearly mentioned in instructions for use.		Enclosed single-use, disposable cartridge
Result display/ interpretation	Result can be read with the naked eye with minimal instructions for interpretation required by user, or with an embedded digital reader with an easy pictorial (e.g., + sign for reactive; - sign for non-reactive; x for invalid) or word display: (reactive, non-reactive, invalid) for each test; no instructions for interpretation required.		Results can be text, symbol or picture format, no user interpretation required
Sample types	Oral fluid/saliva, finger-prick whole blood.	Oral fluid/saliva, finger-prick whole blood. Other fluids not excluded if supported by data.	Plasma or serum. Oral fluid and whole blood not yet tested.

Sample volume	Finger-prick blood, maximum 50μl. Multiple gum swabs or prolonged oral fluid collection (<1 minute).	Finger-prick blood, maximum 10μl. One swab of gums for oral fluid.	6μl plasma sample required; *ca.* 20μl whole blood
Target prices for disposable test strip/cartridge	≤ US$ 1.06	≤ US$ 0.72	< US$ 1.50

Supplementary Table 2: Selected sections from the WHO Target Product Profile for an HIV self-test [3]. The entire set of criteria can be viewed at http://www.idc-dx.org/resources/target-product-profile-hiv-self-test. The self-test criteria were used to indicate the most stringent operational requirements possible for the test described here.

Supplementary References

1. Tomaras, G. D. & Haynes, B. F. HIV-1-specific antibody responses during acute and chronic HIV-1 infection. *Current Opinion in HIV and AIDS* **4,** 373–379 (2009).
2. Armbruster, D. A. & Pry, T. Limit of blank, limit of detection and limit of quantitation. *Clin Biochem Rev* **29 Suppl 1,** S49–52 (2008).
3. PATH. *Target Product Profile: HIV Self-Test Version 4.1: A White Paper on the Evaluation of Current HIV Rapid Tests and Development of Core Specifications for Next-Generation HIV Tests*. 1–60 (2014).
